# Author Correction: Limits of Risk Predictability in a Cascading Alternating Renewal Process Model

**DOI:** 10.1038/s41598-018-19726-y

**Published:** 2018-01-22

**Authors:** Xin Lin, Alaa Moussawi, Gyorgy Korniss, Jonathan Z. Bakdash, Boleslaw K. Szymanski

**Affiliations:** 1 Social and Cognitive Networks Academic Research Center, Rensselaer Polytechnic Institute, Troy, NY 12180 USA; 2 Dept. of Computer Science, Rensselaer Polytechnic Institute, 110 8th Street, Troy, NY 12180 USA; 3 Dept. of Physics, Applied Physics and Astronomy, RPI, 110 8th Street, Troy, NY 12180 USA; 4 US Army Research Laboratory, Aberdeen Proving Ground, MD, 21005 USA

Correction to: *Scientific Reports* 10.1038/s41598-017-06873-x, published online 27 July 2017

The Acknowledgments section of this Article is incomplete.

“This work was partially supported by DTRA Award No.HDTRA1-09-1-0049, by the Army Research Laboratory under Cooperative Agreement Number W911NF-09-2-0053 (the ARL Network Science Collaborative Technology Alliance). The views and conclusions contained in this document are those of the authors and should not be interpreted as representing the official policies either expressed or implied of the U.S. Army Research Laboratory or the U.S. Government”.

should read:

“This work was partially supported by DTRA Award No. HDTRA1-09-1-0049, by the Army Research Laboratory under Cooperative Agreement Number W911NF-09-2-0053 (the ARL Network Science Collaborative Technology Alliance), and by the Army Research Office under grant W911NF-16-1-0524. The views and conclusions contained in this document are those of the authors and should not be interpreted as representing the official policies either expressed or implied of the U.S. Army Research Laboratory or the U.S. Government”.

